# Prevalence of malnutrition in coronavirus disease 19: the NUTRICOV study

**DOI:** 10.1017/S0007114520005127

**Published:** 2020-12-21

**Authors:** Antoine Rouget, Fanny Vardon-Bounes, Pierre Lorber, Adrien Vavasseur, Olivier Marion, Bertrand Marcheix, Olivier Lairez, Laurent Balardy, Olivier Fourcade, Jean-Marie Conil, Vincent Minville

**Affiliations:** 1 Department of Anesthesiology and Critical Care Unit, CHU Toulouse Rangueil, 1 av du Pr Jean Poulhès, 31400 Toulouse, France; 2 INSERM UMR 1048, Institut des maladies métaboliques et cardiovasculaires, CHU Toulouse, 1 av du Pr Jean Poulhès, 31400 Toulouse, France; 3 Department of Radiology, CHU Toulouse Rangueil, 1 av du Pr J Poulhès, 31400 Toulouse, France; 4 Department of Nephrology and Organ Transplant, CHU Toulouse Rangueil, 1 av du Pr Jean Poulhès, 31400 Toulouse, France; 5 Department of Cardiac Surgery, CHU Toulouse Rangueil, 1 av du Pr Jean Poulhès, 31400 Toulouse, France; 6 Department of Cardiology, CHU Toulouse Rangueil, 1 av du Pr Jean Poulhès, 31400 Toulouse, France; 7 Department of Geriatric Medicine, CHU Toulouse Purpan, Place du Dr Baylac, 31000 Toulouse, France

**Keywords:** COVID-19, Malnutrition, Intensive care units, European Society of Parenteral and Enteral Nutrition

## Abstract

Recent European Society of Parenteral and Enteral Nutrition guidelines highlighted the interest of prevention, diagnosis and treatment of malnutrition in the management of coronavirus disease 19 (COVID-19) patients. The aim of our study was to evaluate the prevalence of malnutrition in patients hospitalised for COVID-19. In a prospective observational cohort study malnutrition was diagnosed according to the Global Leadership Initiative on Malnutrition (GLIM) two-step approach. Patients were divided into two groups according to the diagnosis of malnutrition. Covariate selection for the multivariate analysis was based on *P* <0·2 in univariate analysis, with a logistic regression model and a backward elimination procedure. A partitioning of the population was realised. Eighty patients were prospectively enrolled. Thirty patients (37·5 %) had criteria for malnutrition. The need for intensive care unit admission (*n* 46, 57·5 %) was similar in the two groups. Three patients who died (3·75 %) were malnourished. Multivariate analysis exhibited that low BMI (OR 0·83, 95 % CI 0·73, 0·96, *P* = 0·0083), dyslipidaemia (OR 29·45, 95 % CI 3·12, 277·73, *P* = 0·0031), oral intake reduction <50 % (OR 3·169, 95 % CI 1·04, 9·64, *P* = 0·0422) and glomerular filtration rate (Chronic Kidney Disease Epidemiology Collaboration; CKD-EPI) at admission (OR 0·979, 95 % CI 0·96, 0·998, *P* = 0·0297) were associated with the occurrence of malnutrition. We demonstrate the existence of a high prevalence of malnutrition in a general cohort of COVID-19 inpatients according to GLIM criteria. Nutritional support in COVID-19 care seems an essential element.

Coronavirus disease 2019 (COVID-19) caused by the severe acute respiratory syndrome coronavirus-2 appeared in China in December 2019 and is spreading worldwide^(1,2)^. It can evolve to pneumonia requiring hospitalisation up to severe acute respiratory distress syndrome managed in intensive care unit (ICU)^(3)^. Infectious respiratory diseases lead to malnutrition, which can worsen the prognosis^(4,5)^. In COVID-19 population, studies have reported that about half of the patients describe olfactory and gustatory dysfunction^(6–8)^. These disorders may contribute to a reduction in nutritional intakes. Li *et al*. showed a high prevalence of malnutrition (52·7 %) in a cohort of 182 elderly patients with COVID-19^(9)^. Recent European Society of Parenteral and Enteral Nutrition guidelines highlighted the interest of prevention, diagnosis and treatment of malnutrition in the management of COVID-19 patients^(10)^. However, to date there are no data about the prevalence of malnutrition in patients hospitalised for COVID-19.

The aim of our study was to evaluate the prevalence of malnutrition in patients hospitalised for COVID-19.

## Methods

This was a prospective observational cohort study (NUTRI-COV) conducted in Toulouse tertiary hospital from March to April 2020. Approval for the present study (Ethical Committee N^o^ 2020-A01237-32) (RC31/20/0165 NUTRI-COV) was provided by the ‘Comité de Protection des Personnes OUEST I’, France on March 2020. All patients were included in this observational study after verification of informed consent.

### Patients

Eligibility criteria were as follows: (1) age >18 years, (2) severe acute respiratory syndrome coronavirus-2 pneumonia (confirmed by RT-PCR, (3) hospitalisation in wards or ICU, (4) without end-of-life decisions and (5) affiliated to the French national health care system. Patients were excluded if they were pregnant or if they refused to participate.

### Procedures

At admission, patients were examined by medical practitioner according to current recommendations who collected the following data: weight, BMI calculation, recent weight loss, daily oral intake self-reported during the week prior to hospitalisation (the oral intake in the previous week was reported by the patient or his relatives when the patient was not able to express it (ICU, death)), self-reported factors influencing oral intake reduction and Nutrition Risk Screening 2002. For the food intakes, we used the self-evaluation food intake according to Bouette *et al*.^(11)^ which finds a correlation between undernutrition and self-reported food intakes according to a simple analogical visual scale. For anthropometric data (height and weight), patients were weighed on admission. The height was retrieved from previous data if these data were available. When height was not reported during a previous stay, the patient declared his height or it was measured in case of impossibility to declare his height (intensive care).

Factors that may influence oral intake were prospectively recovered on admission in the form of open-ended questions such as: ‘for what reasons did your oral intake decrease in the week prior to your hospitalisation?’ The following items were not proposed: anosmia, dysgeusia, asthenia and dyspnoea in order not to influence the patient. When the patient was unable to answer the questions (ICU, death), the family member who had been closest to the patient and the attending physician were contacted in search of these different elements and the patient questioned as soon as possible to refute or confirm the statements made by the family members when it was feasible.

During the hospitalisation, daily oral intake was recorded by specialist dietitian. Demographic characteristics, co-morbidities, ICU severity score (Simplified Acute Physiology Score II)^(12)^, treatments and biological data were collected from the patient medical record including C reactive protein, albuminaemia, protidaemia, serum creatinine, glomerular filtration rate (Chronic Kidney Disease Epidemiology Collaboration (CKD-EPI) formula), lymphocyte count and D-dimers at admission. Data collection was done prospectively by a third-party physician not involved in the patient’s care.

Malnutrition was diagnosed according to the Global Leadership Initiative on Malnutrition (GLIM) two-step approach defined by the association of one phenotypic criterion (especially non-volitional weight loss, low BMI) and one aetiological criterion (reduced food intake or assimilation, disease burden/inflammatory condition)^(13)^.

Due to COVID-19, it was recommended not to use conventional methods for assessing lean body mass in patients because of the risk of contamination for caregivers. All patients undergoing chest CT scan were assessed for an association between undernutrition and pectoral muscle area assessment of muscle mass. This index is not used in nutritional assessment and we wanted to study this index in a pilot way in undernutrition in ICU. There are no data on validity or reliability for pectoral muscle area in nutritional assessment.

To estimate muscle mass, the pectoralis muscle area was analysed; the pectoral muscle area is used in pneumology as a prognosis factor^(14)^. Based on chest CT scans which were routinely performed as part of COVID-19 management, pectoralis muscle area was measured by a trained radiologist. For comparability, pectoralis muscle area was indexed to body surface area (pectoralis muscle index) (expressed in cm^2^/m^2^)^(15)^. The section used to evaluate the cross-sectional area of the pectoral muscle was selected by scrolling the scanner towards the pulmonary apex and identifying the first axial image above the aortic arch. The pectoral muscle area (cm^2^) corresponds to the combination of the area of the small pectoral and large right pectoral muscle measured manually using the region of interest (ROI) surface area measurement tool. Indeed, the muscle area is used in oncology and pneumology as a prognostic factor.

### End-points

The primary end-point of the study was the prevalence of malnutrition defined by the number of malnourished patients on the total number of studied patients.

The secondary end-points were the prevalence of severe malnutrition (BMI < 17 kg/m^2^ and/or >10 % non-volitional weight loss in 1 month and/or albuminaemia <30 g/l and/or Nutrition Risk Screening 2002 ≥ 5), pectoralis muscle index on chest CT scan and the association between malnutrition and outcome (need for ICU admission, hospital and ICU length of stay, mechanical ventilation duration, in-hospital mortality). In order to differentiate between severe malnutrition, we used the French recommendations of the Haute Autorité de Santé^(16)^. These recommendations overlap with the GLIM recommendations but have the particularity of adding deep hypoalbuminaemia (<30 g/l) as a severity criterion. The severity of undernutrition influences the level of surveillance and nutritional management.

### Statistical analysis

First, variables distribution was verified with Shapiro–Wilk test. Patients were divided into two groups according to the diagnosis of malnutrition. Then, data are presented as medians and interquartile ranges (IQR) or means and standard deviations. Categorical data are expressed as numbers and percentages.

To compare the different parameters, parametric and non-parametric tests were used as appropriate (*t* test or Mann–Whitney for continuous variables and *χ* ^2^ or Fisher’s exact test for categorical variables).

Covariate selection for the multivariate analysis was based on *P* <0·2 in univariate analysis, with a logistic regression model and a backward elimination procedure. For the validation of the selected model, we used the Hosmer and Lemeshow (goodness of fit test), the percentage of prediction of the model and its AUC. To highlight covariates associated with malnutrition, a partitioning of the population was represented using a Classification and Regression Tree analysis. The advantage of this multivariate analysis approach is to describe the means of distribution of the population in homogeneous groups according to the existence of malnutrition and the covariates selected from the multidimensional analysis.

Statistical analyses were conducted using SPSS® for Window version 24 (IBM Corporation). *P* ≤0·05 was considered statistically significant.

## Results

### Characteristics of the population

From March to April 2020, eighty patients were prospectively enrolled in the study. We have included all patients responding to the inclusion criteria for the given period in our hospital. No patients withdrew their consent or refused to participate.

The baseline characteristics of the patients are shown in Table 1. Seventeen patients (21·2 %) were over 70 years old. The median time from the onset of COVID-19 symptoms to hospitalisation was 7 (IQR 5·5–11) d. Patients reported dysgeusia (27·8 %), anorexia (27·8 %), asthenia (21·5 %) and anosmia (20·3 %). Thirty-seven (46·2 %) declared a food intake below 50 %.

**Table 1.** Baseline characteristics of the population (Medians and 25th–75th percentiles; number and percentages; ranges)

	General characteristics of the population at admission (*n* 80)		
	Median	25–75 percentiles	Range
Demographic characteristics			
Age (years)	59·5	49·5 to 68·5	19 to 87
Sex ratio			
Male			
*n*	60		
%	75		
Female			
*n*	20		
%	25		
SAPS II	36	29 to 44	8 to 76
In-hospital mortality			–
*n*	3		
%	3·75		
Basis weight (kg)	87·5	76 to 100	61 to 135
Weight at admission (kg)	83	72 to 95·5	58 to 130
Absolute weight loss (kg)	−3·8	−6– to −2	0 to −9·3
BMI (kg/m^2^)	28·3	25 to 31	20·4 to 46·8
>50% oral intake reduction*			–
*n*	37		
%	46·2		
NRS-2002	4	3 to 5	2 to 7
Medical history			
Active smoking			–
*n*	9		
%	11·3		
Diabetes			–
*n*	24		
%	30		
Dyslipidaemia			–
*n*	8		
%	10		
Hypertension			–
*n*	34		
%	42·5		
Organ transplantation			–
*n*	10		
%	12·5		
Onco-haematological disease			–
*n*	10		
%	12·5		
Paraclinical parameters			
Albuminaemia (g/l) (*n* 65)	24	18 to 27	12 to 39
Prealbuminaemia (g/l) (*n* 56)	0·12	0·085 to 0·22	0·03 to 0·76
CRP (mg/ml) (*n* 75))	88	43·8 to 133·4	2·5 to 403
Serum creatinine (µmol/l)	83·5	66·5 to 122	36 to 680
GFR (ml/min per 1·73 m^2^)	82	53 to 100	5 to 118
Kaliaemia (mmol/l)	3·5	3·3 to 3·7	2·5 to 4·5
Phosphataemia (mmol/l) (*n* 66)	0·77	0·61 to 0·94	0·29 to 1·35
Magnesaemia (mmol/l) (*n* 51)	0·8	0·5 to 1·31	0·5 to 1·31

SAPS II, Simplified Acute Physiology Score; NRS-2002, Nutrition Risk Screening; CRP, C-reactive protein; GFR, glomerular filtration rate.

* 7 d before admission.

### Primary outcome

Thirty patients (37·5 %) had criteria for malnutrition of which 21/30 (70 %) met criteria for severe malnutrition. The comparison between patients with malnutrition or not is presented in Table 2. They were comparable except for the glomerular filtration rate (CKD-EPI) at arrival, more impaired in the malnutrition group (71·5 (IQR 41–91) *v.* 85·5 (IQR 64–101) ml/min per 1·73 m^2^).

**Table 2.** Comparison between patients with malnutrition and no malnutrition (Medians and 25th–75th percentiles; number and percentages)

	No malnutrition (*n* 50)		Malnutrition (*n* 30)		*P*
	Median	25th–75th percentiles	Median	25th–75th percentiles	
Demographic characteristics					
Age (years)	57	49 to 65	63·5	53 to 69	0·116
Sex ratio					0·427
Male					
*n*	32		24		
%	72		80		
Female					
*n*	14		6		
%	28		20		
SAPS II	37	29 to 47·5	34	26 to 43	0·499
In-hospital mortality					0·049†
*n*	0		3		
%	0		10		
Basis weight (kg)	89·5	79 to 102	86·9	73 to 99	0·253
Weight at admission (kg)	87·3	76 to 100	80·9	67 to 92	0·029†
Absolute weight loss (kg)	−3	−3 to −1·5	−6	−7 to −6	< 0·0001†
BMI (kg/m^2^)	28·4	25·8 to 32·8	26·4	22·8 to 30·6	0·033†
>50% oral intake reduction*					0·058
*n*	19		18		
%	38		60		
NRS-2002	4	3 to 5	5	4 to 5	0·026†
Medical history					
Active smoking					0·999
*n*	6		3		
%	12		10		
Diabetes					0·108
*n*	12		12		
%	24		40		
Dyslipidaemia					0·0185†
*n*	2		6		
%	4		20		
Hypertension					0·477
*n*	20		14		
%	40		46·7		
Organ transplantation					0·485
*n*	5		5		
%	10		16·7		
Onco-haematological disease					0·16
*n*	4		6		
%	8		20		
COVID-19 characteristics					
Delay between onset of symptoms and hospitalisation (d)	7·5	6 to 11	7	5 to 11	0·671
Dysgeusia					0·130
*n*	11		11		
%	22		36·7		
Anosmia					0·516
*n*	9		7		
%	18		23·3		
Anorexia					0·633
*n*	13		9		
%	26		30		
Asthenia					0·206
*n*	13		4		
%	26		13·3		
Paraclinical parameters					
Albuminaemia (g/l)	25	17 to 29·3	20·5	18 to 25·5	0·162
Prealbuminaemia (g/l)	0·11	0·07 to 0·24	0·13	0·09 to 0·19	0·689
CRP (mg/ml)	87	39· to 128	96	5 to 154·8	0·414
Serum creatinine (µmol/l)	80·5	63 to 107	101·5	69 to 153	0·060
GFR (ml/min per 1·73 m^2^)	85·5	64 to 101	71·5	41 to 91	0·020†
Pectoralis muscle area (cm^2^)	22·35	16·4 to 27·6	22·2	16 to 28·6	0·768
Pectoralis muscle index (cm^2^/m^2^)	7·401	5·81 to 9·3	7·448	4·88 to 9·64	0·644

SAPS II, Simplified Acute Physiology Score II; NRS-2002, Nutrition Risk Screening; COVID-19, coronavirus disease 19; CRP, C-reactive protein; GFR, glomerular filtration rate.

* 7 d before the admission.

† *P* < 0·05 is significant.

### Secondary outcomes

Twenty-one (26 %) patients met criteria for severe malnutrition. Nutrition Risk Screening 2002 was more elevated in malnourished patients (5 (IQR 4–5)) in comparison with patients without malnutrition (4 (IQR 3–5)) (*P* = 0·026). Serum albumin concentration was 25 (IQR 17–29·3) g/l in the group without malnutrition *v*. 20·5 (IQR 18–25·5) g/l in the malnutrition group (*P* = 0·162). Hypoalbuminaemia (<30 g/l) was similar between the two groups (75·6 % (*n* 31) *v.* 91·7 % (*n* 22), *P* = 0·11), respectively, in the group without malnutrition in comparison with malnutrition group. Pectoralis muscle index was not different between the groups without malnutrition *v.* the malnutrition group (respectively 7·40 (IQR 5·81–9·3) *v.* 7·448 (IQR 4·88–9·64) cm^2^/m^2^, *P* = 0·644).

The need for ICU admission (*n* 46, 57·5 %) was similar in the two groups, respectively 56 % (*n* 28) in the no malnutrition group *v.* 60 % (*n* 18) in the malnutrition group (*P* = 0·72). There was not statistical difference between mechanical ventilation duration, ICU and hospital lengths of stay between the two groups.

Among the eighty patients included in the study, thirty (37·5 %) presented malnutrition. Three patients who died (3·75 %) were malnourished. Multivariate analysis exhibited that low BMI (OR 0·83, 95 % CI 0·73, 0·96, *P* = 0·0083), dyslipidaemia (OR 29·45, 95 % CI 3·12, 277·73, *P* = 0·0031), oral intake reduction <50 % (OR 3·169, 95 % CI 1·04, 9·64, *P* = 0·0422) and glomerular filtration rate (CKD-EPI) at admission (OR 0·979, 95 % CI 0·96, 0·998, *P* = 0·0297) were associated with the occurrence of malnutrition in COVID-19 inpatients (Table 3).

**Table 3.** Multivariate analysis* (Odds ratios and 95 % confidence intervals)

Significant covariates	OR	95 % CI	*P*
BMI (kg/m^2^)	0·8363	0·73, 0·96	0·0083†
Dyslipidaemia	29·4532	3·12, 277·73	0·0031†
Oral food intakes 7 d before admission <50 %	3·1687	1·04, 9·64	0·0422†
GFR (CKD-EPI) (ml/min per 1·73 m^2^)	0·9785	0·96, 0·998	0·0297†

GFR, glomerular filtration rate; CKD-EPI, Chronic Kidney Disease Epidemiology Collaboration.

* Hosmer and Lemeshow test 0·7; model prediction percentage 77 %; AUC 0·82 (0·72, 0·898).

† *P* < 0·05 is significant.

**Fig. 1.**
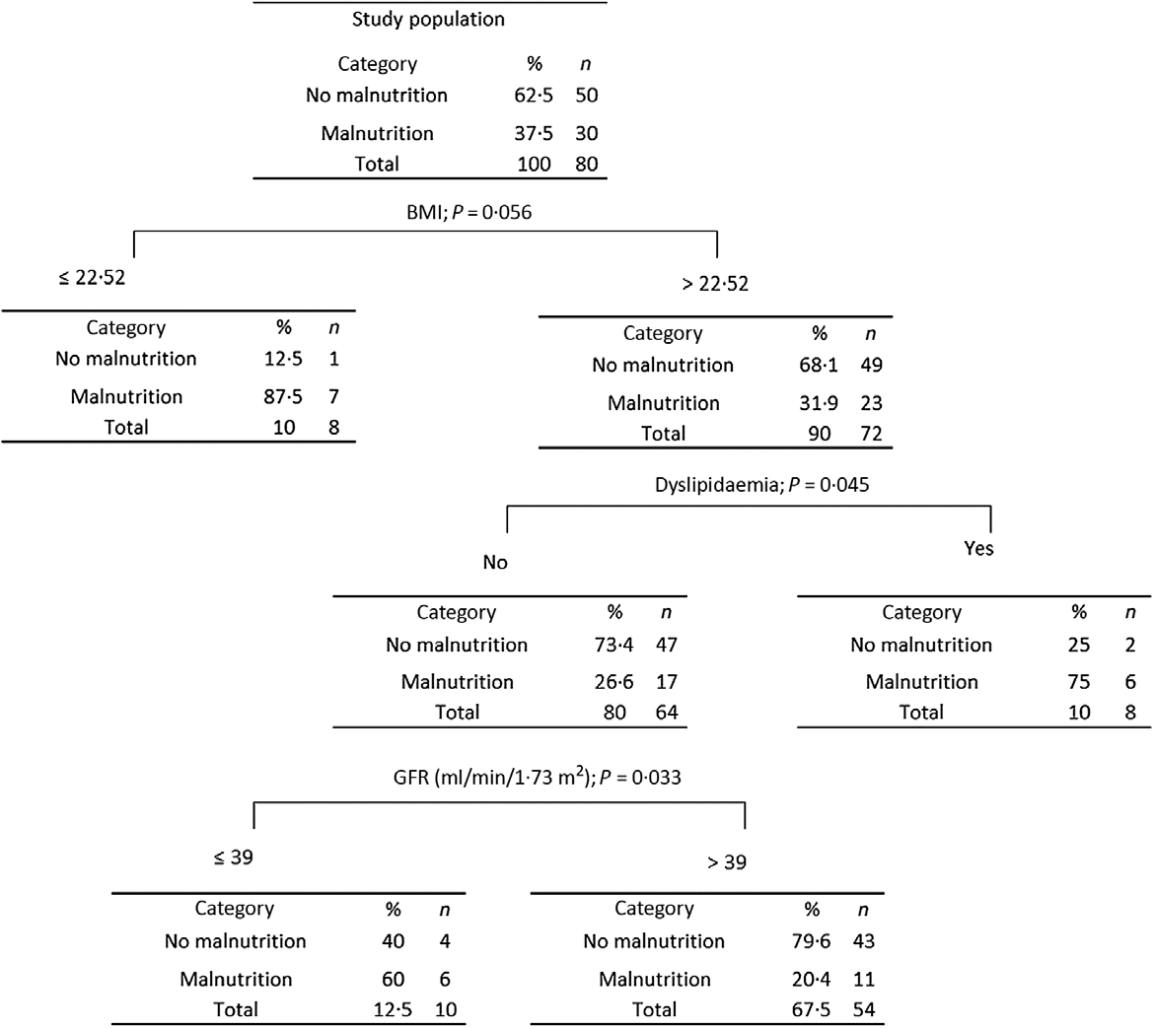
Classification and Regression Tree (CART) according to BMI, dyslipidaemia and glomerular filtration rate (GFR).

Results of Classification and Regression Trees are shown in Fig. 1. Classification and Regression Tree analysis has been used extensively as an alternative to the classical linear and additive prediction models.

Results are presented in the form of a tree with a hierarchical sequential structure. The segmentation (multivariate analysis) shows the most important variables in homogeneous groups according to malnutrition. The percentage of estimation of this Classification and Regression Tree analysis was 80 %. For example, patients with BMI ≤ 22·5 kg/m^2^ presented a malnutrition status in 87·5 % of the cases. In contrast, patients with BMI > 22·5 kg/m^2^ and with dyslipidaemia were malnourished in 75 % of the cases. In case of absence of dyslipidaemia and if glomerular filtration rate is ≤39 ml/min per 1·73 m^2^, 60 % of the patients were malnourished.

## Discussion

To our knowledge, the present study is the first reporting the prevalence of malnutrition in patients hospitalised for severe acute respiratory syndrome coronavirus-2 pneumonia. It showed a high prevalence (37·5 %) of malnutrition in hospitalised patients with 26 % of severe malnutrition. However, we have probably overestimated the prevalence of severe undernutrition in our patients using hypoalbuminaemia as recommended by the French guidelines; we believe that this hypoalbuminaemia is more reflective of the inflammatory state of these patients^(16)^.

Median albuminaemia was very low (24 (IQR 18–27) g/l) and pectoralis muscle area index was not associated with recent malnutrition. There was no association between recent malnutrition and the need for ICU admission. However, there were more deaths in the malnutrition group. The included population was comparable to recent publications of COVID-19 cohorts in terms of age, sex ratio^(17)^ and co-morbidities (BMI, diabetes mellitus, hypertension)^(18)^. The population of the study was overweight (median BMI 28·5 (IQR 25–31) kg/m^2^) consistent with numerous previous studies^(19,20)^.

A recent Chinese publication found a high prevalence (52·7 %) of malnutrition in 182 elderly patients with COVID-19^(9)^ diagnosed with the Mini Nutritional Assessment^(21)^. The mean age of the population was 68·5 years old. Interestingly, in this younger cohort (59·5 (IQR 49·5–68·5) years old), we described more than one-third of malnourished patients (37·5 %). The diagnosis of malnutrition was based on international GLIM criteria^(21)^, not specific to an elderly population and easy to use. All hospitalised patients got the aetiological criterion (pneumonia), the phenotypic criterion being based solely on a recent weight loss of 5 % in our overweight population.

Concerning the weight loss and the importance of fasting, almost 46 % (*n* 37) of the patients reported decreased food intakes with multiple reasons. They mentioned anorexia (27·5 %), asthenia (21·25 %), dysgeusia (27·5) and anosmia (20 %). Surprisingly, no statistical association was made between the self-reported importance of starvation and the existence of malnutrition. In a recent publication, Bouëtté *et al.* found an association between oral intakes <7/10 and the existence of malnutrition according to GLIM criteria in a population of general medicine practice patients^(11,13)^. One first explanation of our results could be a lack of power for this criterion. Another explanation could be related to the inflammatory nature of COVID-19 malnutrition. In ninety-seven patients, Hedlund *et al*. found an association between hypoalbuminaemia, inflammation and outcome in patients hospitalised for community-acquired pneumonia^(22)^. The authors argue that hypoalbuminaemia is explained by the inflammatory status more than their nutritional status^(22)^.

It should be noted that the nutritional assessment was conducted at admission, with a median onset of COVID-19 symptom of 7 d. Thus, we highlighted an acute malnutrition. The concept of acute malnutrition is described and might need a specific management^(23)^.

Concerning metabolic disorders, hyperlipidaemia affects immune functions and could promote COVID-19 susceptibility^(24)^. Hypercholesterolaemia is associated with cholesterol accumulation in immune cells, which participate to inflammatory responses and may affect the response to infections^(25)^. Our multivariate analysis found an association between malnutrition and dyslipidaemia in this context of inflammatory disease. To our knowledge, there are no data that can specifically explain this association.

Pectoralis muscle index has been suggested as a prognostic marker in relation to muscularity in an oncology population^(26)^. In our population, there was no difference in this index between malnourished and non-malnourished patients. This could be explained by the recent development of this malnutrition. There was no statistical association between the pectoralis muscle index and patients’ outcomes.

The three patients who died (3·75 %) were malnourished. One of them died after withdrawal of life-sustaining measures. The others, with extracorporeal life support, died from severe intracranial haemorrhage. Our study was not designed to analyse this association. Association between nutritional status and outcome is well known^(27,28)^. In contrast, there was no association between malnutrition and the need for ICU admission or hospital length of stay in our study.

### Strengths and weaknesses

NUTRICOV strengths are the prospective design of the study, which allowed for an exhaustive collection and the use of international tools (GLIM definition and Nutrition Risk Screening 2002). Therefore, this is the first study analysing malnutrition in a general population of COVID-19 inpatients. The limits are the declarative nature of some collected data (basis weight, oral intakes prior to hospitalisation). Another potential bias is the measurement of the height of prone patients in ICU, which is less reliable than a vertical measurement. This might have influenced our BMI calculations. We used GLIM criteria to define malnutrition. GLIM definition may lead to higher prevalence because of requiring fewer criteria in comparison with European Society of Parenteral and Enteral Nutrition definition. In a recent publication, Clark *et al*. exhibited a small agreement between GLIM and European Society of Parenteral and Enteral Nutrition definition for malnutrition^(29)^.

### Conclusion

Severe acute respiratory syndrome coronavirus-2 is responsible for severe forms of pneumonia requiring hospitalisation. European Society of Parenteral and Enteral Nutrition recently suggests the existence of a nutritional risk and recommends to routinely check for malnutrition in COVID-19 patients to improve global patient care. We demonstrate the existence of a high prevalence of malnutrition (37·5 %) in a general cohort of COVID-19 inpatients according to GLIM criteria. Considering this high prevalence, nutritional support in COVID-19 care seems an essential element.
